# Supplementary material for: Content-rich biological network constructed by mining PubMed abstracts
Source: BMC Bioinformatics. 2004 Oct 8;5:147. doi: 10.1186/1471-2105-5-147 (PMC528731; doi:10.1186/1471-2105-5-147)
Supplement: Additional File 2 — The original results of the above study (non-essential files are deleted to keep the file size under the limit set by BMC bioinformatics). [file 1471-2105-5-147-S2.bz2 › chilibotAdditionalFile2/dip10/35ID8530384E122/html/POU2F2_POU2AF1.html]

 


 **POU2F2** and **POU2AF1** 
  
Found 53 abstracts in PubMed, retrieved 10.  
 

 What does Google say? 
 PDF only 
| .edu only 

---

**Interactive relationship** (e.g. stimulation, inhibition, etc)

**Neutral relationship**- These findings support the notion that Oct 2  [ **POU2F2** ]  regulates gene transcription by both OBF 1  [ **POU2AF1** ]  dependent and independent mechanisms.  Ref: 11937630 Nucleic Acids Res, 2002
- Our data indicate that the PORE type Oct1 or Oct2  [ **POU2F2** ]  dimer, rather than the monomer, is the primary target of the cofactor OBF1  [ **POU2AF1** ] .  Ref: 12727885 EMBO J, 2003
- Oct 2  [ **POU2F2** ]  regulates CD36 gene expression via a consensus octamer, which excludes the co activator OBF 1  [ **POU2AF1** ] .  Ref: 11937630 Nucleic Acids Res, 2002
- BOB  [ **POU2AF1** ] .1 OBF  [ **POU2AF1** ] .1 also called OCA B  [ **POU2AF1** ] , a B lymphocyte specific transcriptional coactivator, is recruited to octamer containing promoters by interacting with the Oct 1 or Oct 2  [ **POU2F2** ]  proteins.  Ref: 12207332 Eur J Immunol, 2002

**Non-interactive relationship** (e.g. studied together, co-existance, homology, etc.)

- Our data also demonstrate that CD36 is the first example of an Oct 2  [ **POU2F2** ]  dependent gene whose expression in B cells is independent of OBF 1  [ **POU2AF1** ] .  Ref: 11937630 Nucleic Acids Res, 2002
- The transcription factors BSAP, BOB  [ **POU2AF1** ] .1, Oct2  [ **POU2F2** ]  and MUM1 are sequentially expressed in normal B cell development.  Ref: 12647220 Virchows Arch, 2003
- The expression patterns of BCL6, CD138, Oct2  [ **POU2F2** ] , and BOB1  [ **POU2AF1** ]  in HRS cells of lrcHL showed differences to those of both cHL and lpHL.  Ref: 12670918 Cancer Res, 2003
- This inactivity correlates with either reduced levels or even a complete lack of several B cell specific transcription factors required for their expression Oct 2  [ **POU2F2** ] , OBF 1  [ **POU2AF1** ] , PU.1, E47 E12, PAX 5 and EBF.  Ref: 12118370 Oncogene, 2002
- On immunohistochemistry, the following phenotype was observed CD45, CD20, CD79a, PAX5 BSAP, BOB  [ **POU2AF1** ] .1, Oct 2  [ **POU2F2** ] , PU.1, Bcl 2, CD30, HLA DR, MAL protein, Bcl 6, MUM1 IRF4, CD10, CD21, CD15, CD138, CD68, and CD3.  Ref: 12507907 Am J Pathol, 2003
- it is closely related to the mixed cellularity CHL MCHL in respect of BSAP, BOB  [ **POU2AF1** ] .1, and Oct2  [ **POU2F2** ]  expression.  Ref: 12647220 Virchows Arch, 2003
- However, it differs from other aggressive B cell lymphomas in that it shows defective immunoglobulin production despite the expression of OCT 2  [ **POU2F2** ] , BOB  [ **POU2AF1** ] .1, and PU.1 transcription factors and the lack of IgV gene crippling mutations.  Ref: 12507907 Am J Pathol, 2003
- B lymphoma cell lines have been generated from mice with targeted mutations in the oct 2  [ **POU2F2** ] , OBF 1  [ **POU2AF1** ] , vav 1 and btk genes, as a model system that lacks these limitations and possesses additional potential for experimental manipulation.  Ref: 12848851 Immunol Cell Biol, 2003
